# The Obstetric Memoirs and Contributions of James Y. Simpson, M. D., F. R. S. E., Professor of Midwifery in the University of Edinburgh, Etc., Etc.

**Published:** 1856-09

**Authors:** 


ART. VI.—*The Obstetric Memoirs and Contributions of James Y. Simpson, M. D., F. R. S. E., Professor of Midwifery in the University of Edinburgh, etc., etc.* Edited by W. O. Priestly, M. D., Edinburgh, and Horatio R. Storer, M. D. Boston, U. S. Volume II. Philadelphia: J. B. Lippincott & Co. 1856.

This volume, like that which preceded it some months since, and which we then mentioned in terms of warm commendation, is made up of the fugitive papers of a busy practitioner. It is an indication of what may be accomplished by an earnest mind with trained habits of industry. We know of no one fact which should more stimulate our industry, or reproach our indolence, than the publication of these splendid octavos.

The series of papers included in this second volume supply many deficiencies left by the first; but, as in its predecessor, every subject attempted is thoroughly treated in an exhaustive manner. Four groups of subjects receive attention, viz:

The Pathology of the Puerperal State;

The Physiology and Pathology of the Products of Conception;

The Pathology of Infancy and Childhood; and

Anæsthetics in Midwifery, Surgery, etc.

The arrangement is comprehensive, and while this is no text-book for the student, it will be a *vade-mecum* for the practitioner. It is precisely that sort of volume which he may call in consultation with an assurance of finding just what he wants.

The subject of puerperal fever is the first in order. Dr. Simpson advocates most ably those opinions which regard it as an eminently contagious disease, and with that tendency to generalization which marks his mind, couples with it surgical fever, as but another form of the same manifestation. In this connection we beg our readers to refer to the case of Dr. Potter, of Geneva, to be found in our editorial department, and to read it in connection with the following extract from the work under review. There can be no doubt that surgeons are in many instances the vehicle of fatal disease, and we shall endeavor to draw attention to this important feature in the history of contagion:

"Dr. Arneth's very valuable paper adduced what was apparently incontrovertible evidence of puerperal fever being propagated in the way he suggested, viz., by medical men carrying on their fingers matter capable of producing it from bodies which they were dissecting, and inadvertently inoculating that matter into the mucous membrane of the vagina of patients in labor. In these cases, the fingers of the accoucheur when once dipped in the poison might retain it, till they had again inoculated that poison into the bodies other healthy subjects. The vaginal mucous membrane was generally stretched and abraded in labor; the perineum was often slightly torn; and the whole afforded a surface in a condition easily inoculable. But if students and practitioners, with their hands containing some portion of morbid matter, can thus, by inoculating that matter on the abraded surface of the vagina, produce puerperal fever, no doubt, under similar circumstances, surgeons could and did inoculate into the wounds which they made or dressed, similar matter producing the similar disease of surgical fever in their patients. If it could be inoculated into the abraded surface of the vagina, it could be inoculated into a recent wound. If it produced fever in the one set of patients, it would produce fever in the other. And since bringing under the attention of the profession the communicability of surgical fever, Dr. Simpson stated, that he had heard various facts in regard to it, all of which more and more convinced him that surgeons, like accoucheurs were occasionally the unhappy media of inoculating their patients with morbid matter, producing in them surgical fever, as in puerperal patients, obstetricians, by the same means, produced in their patients puerperal fever. He had no doubt that it would take many long years fully to convince surgeons of this fact; but still it was his conviction, that surgeons would ultimately both believe and act upon it, and that their doing so would be a means of preventing many of the numerous deaths which now occur after operations, particularly in hospital surgical practice."

Following this we have a most interesting and original article on "puerperal arterial obstruction and inflammation." A long series of cases is given. First, we have a class of cases which depend on the detachment of cardiac vegetations, and their propulsion from the cavities of the heart into the vessels. Dr. Simpson gives several cases in which yellow, fibrinous clots were found in the common iliac arteries, whither they had doubtless been borne by the current of the circulation from the heart, and other cases of sudden puerperal death, when the obstruction was found in the pulmonary arteries. We have ourself seen cases of the latter form, not in puerperal women, but in men subject to syncope. The pathology is simple. A rough projection or growth on the endocardium affords the nucleus of a clot, which syncope favors; presently it becomes detached, and, floating into the pulmonary artery, causes sudden death, or passing down the aorta blocks up the common iliacs and produces gangrene. Under this head, also, Prof. Simpson includes cases of spontaneous arterial laceration, of phlebitic pneumonia and puerperal phlebitis. We would gladly give a summary of the highly practical essays on these and kindred subjects, but must pass to puerperal tetanus. Prof. S. regards cold as the more common cause of this terrific disease—terrific, though less fatal in women than in men. On the subject of treatment we have only space for the following extract:

"1st. The greatest possible quietude and isolation of the patient from all irritation, corporeal or mental, during the course, and for some time even after the resolution, of the disease.

"2d. The special avoidance of painful and generally impracticable attempts at opening the mouth in order to swallow; but sustaining the strength of the patient, and allaying thirst by enemata, or by fluids applied to the general surface of the body.

"3d. If there is any well-grounded hope of irritating matters lodged in the bowels, acting as an exciting or aggravating cause, to sweep out the intestinal canal at the commencement of the disease with an appropriate enema.

"4th. To relax the tonic spasms of the affected muscles, and diminish the exalted reflex excitability of the spinal system by sedatives, or antispasmodics, with the prospect of either directly subduing this morbid reflex excitability, or of warding off the immediate dangers of the disease, and allowing the case to pass on, from an acute and dangerous attack, to a subacute, and far more hopeful and tractable form of the malady.

"Various sedatives and antispasmodics have been recommended to fulfill this last most vital and important indication in the treatment of tetanus—as belladonna, stramonium, hemlock, henbane, musk, camphor, Indian hemp, hydrocyanic acid, valerian, &c. Perhaps the two drugs of this class that have hitherto been most used, and most relied upon, are opium by the mouth, and tobacco by enema. But certainly we have no decided evidence of the beneficial effects of opium, even in the most heroic doses; and it seems doubtful even if this and other such medicines are readily or at all absorbed from the stomach and upper part of the intestinal canal in cases of acute tetanus. Tobacco in the form of enema has doubtless often acted most favorably in arresting the spasms; but it is a drug, the action of which is not easily or safely kept up with that degree of constancy which is required in acute tetanus. Latterly, the antispasmodic action of sulphuric ether and chloroform has been repeatedly employed to allay that exalted state of the reflex nervous system, and to relax that resulting tonic contraction of the maxillary and other muscles, which constitutes the essence of tetanus. Medical men may yet discover therapeutic agents, to be introduced into the body by inhalation or otherwise, the action of which will be as directly anti-tetanic in their effects, as strychnine is directly tetanic in its properties; and such agents, if they were otherwise innocuous, would form the proper remedies for tetanus. Here, as elsewhere, in future, physicians will probably seek for therapeutic remedies in the same way, and upon the same principles, as toxicologists search for antidotes to poisons. Chloroform in sufficient doses acts as a direct sedative upon the reflex nervous system, and upon exalted muscular contractility. In consequence of this action, it affords us one of the surest and most manageable means of allaying common convulsive attacks; and it has now also, according to the reports in periodical medical literature, been repeatedly successful in the treatment of traumatic tetanus,* whilst it has apparently also repeatedly failed in subduing the more acute forms of the disease. Perhaps some of the failures have arisen from the patient not being kept sufficiently deeply and continuously under the action of the drug. If used in tetanus, its action will require to be sustained for many hours, or oftener, perhaps, for many days. And there is abundant proof of the safety with which its continuous action may be kept up under proper care and watching. For instance, a few months ago I saw, with Dr. Combe, a case of convulsions of the most severe and apparently hopeless kind in an infant of six weeks. The disease at once yielded, and ultimately altogether disappeared, under the action of chloroform, which required to be used almost continuously for thirteen days; as much as 100 ounces of the drug being used during the period. After all tendency to convulsions at last ceased, the little patient rapidly grew, and is at the present moment a very strong, healthy child. In a case of the successful treatment of traumatic tetanus, by Dr. Dusch, above sixty ounces of chloroform were employed.†"

We need only add to this that the use of chloroform in convulsive disease is but imperfectly practiced. Our colleague, Prof. White, was the first in the country. and perhaps in the world, to resort to it in puerperal convulsions; and since that time he has not hesitated to employ it in infantile convulsions. The fact has greater influence with us, knowing as we do that Prof. W. is, in his ordinary obstetric practice, very sparing in the use of this drug. It is no hobby with him.

A variety of other puerperal diseases are considered in this section, but we must pass rapidly on and turn to the "physiology and pathology of the products of conception." The article on the attitude and positions of the fœtus in utero, strikes us as practically useful. It is lengthy and exhaustive.

Among the less practical portions of this section, the reader will notice with admiration the able article on hermaphroditism, to which more than a hundred pages are given. The author, throwing aside the prejudices of medical belief, gives to hermaphroditism a real existence, and supports his position by unanswerable argument. The celebrated case in the possession of Prof. Ackley, of Cleveland, receives notice only in an appendix by the

---

* From Edin. Monthly Journal of Medical Science, January, 1848, p. 545, reported from a discussion in the Medico-Chir. Society.

† Ranking's Abstract, vol. xvii., p. 63.

American editor. He gives, there, the brief statement of Dr. J. B. S. Jackson, of Boston, opposing Dr. Ackley's belief of a complete double hermaphroditism. He does not regard the ovaries as such, though one would suppose that the fact of regular menstruation (which we believe to be well authenticated) was rather more conclusive than an imperfect examination of a specimen which had been several years in alcohol.

The remainder of the volume is devoted to important papers on various diseases of the placenta, of the fœtus in utero, and of infancy and childhood. The valuable article with which many of our readers are familiar, "On the External Use of Oil in the Prevention and Treatment of Scrofula, Phthisis, etc.," is here introduced in a form considerably enlarged and perfected.

The article on anæsthesia is one of the most important in the volume. Prof. Simpson goes to far greater lengths in the use of chloroform than we would sanction; but he is excusable as its introducer and champion against a violent opposition. We quote some general remarks on its exhibition:

"To produce the complete anæsthetic and soporific effects of the chloroform, some conditions are necessary to be attended to.* Without attending to these conditions you will have failures.

"1. The chloroform vapor must always be exhibited as rapidly, and in as full strength as possible, if you desire to have its first or exhilirating stage practically done away with, and excluded; and you effect this by giving the vapor so powerfully and speedily as to apathize the patient at once. If you act otherwise, and give it in small or slow doses, you excite and rouse the patient in the same way as if nitrous oxide gas were exhibited.

"2. In order that the patient be thus brought as speedily as possible under its full influence, the vapor should be allowed to pass into the air-tubes by both the mouth and nostrils—and hence all compression of the nostrils, &c., is to be avoided.

"3. The vapor of chloroform is about four times heavier than atmospheric air. And hence, if the patient is placed on his back during its exhibition, it will, by its mere gravitation, force itself in larger quantities into the air-passages than if he were erect or seated.

"As to the best instrument for exhibiting the chloroform with these indications, the simple handkerchief is far preferable to every means yet adopted. It is infinitely preferable to any instrument I have yet seen, some of which merely exhibit it by the mouth, and not by the nostrils, in small and imper-

---

* See, for example, Dr. Ranking's Abstract, vol. ix., p. 239 (three successful cases); British and Foreign Medical Review for 1851, p. 464 (two successful cases), &c.

fect, instead of full and complete doses; and with instruments so constructed, there is no doubt whatever that failures and exciting effects would ever and anon occur. Besides, inhaling instruments frighten patients, whilst the handkerchief does not; and mental excitement of all kinds, from whispering and talking around the patient, is to be strictly avoided, if possible. As to the quantity required to be applied to the handkerchief, it has been stated, that the average dose of a fluid drachm is generally sufficient to affect an adult; but I have latterly seldom measured the quantity used. We must judge by its *effects*, more than by its quantity. The operator gathering his handkerchief into a cup-like shape in his hand, should wet *freely* the bottom of the cup (so to speak), and if the patient is not affected in a minute or so, he should add a little more. It evaporates rapidly; and you must not wet your handkerchief, and then delay for a minute or more in applying it. It must be applied immediately. Not unfrequently, when the patient is just becoming insensible, he will withdraw his face, or forcibly push aside the handkerchief. If you *then* fail to reapply it to his face, and keep it there, you will be liable to leave him merely excited. But probably two or three inhalations more will *now* render him quite insensible. The simplest test of its full and perfect effect, is some noise or stertor in the respiration. Cease it as soon as this is fully set in. But reapply it, of course, from time to time, if it is wished to keep up its effects."

In this hasty sketch of the second volume of Simpson's Obstetric Works, we have endeavored rather to impress our readers with its value, than to give an analytical review. Withholding our assent from a portion of the author's opinions on hermaphroditism, and deprecating the free use of chloroform in natural labor, we are still anxious that the volume before us should have a large circulation.

It will disappoint him who expects a text-book, but for the intelligent practitioner, who needs a practical work and is willing to forgive the absence of many things which are demanded in a text-book, for the sake of those enlarged views which require space and amplification, we regard it as a most valuable addition to the library.

